# Practice as research as a decolonial praxis: Yoruba culture retrieval

**DOI:** 10.3389/fsoc.2025.1579227

**Published:** 2026-01-09

**Authors:** Lara Rose

**Affiliations:** Leeds Arts Research Centre, Leeds Beckett University, Leeds, United Kingdom

**Keywords:** Yoruba, methodology, practise as research, autoethnography, case studies, art, afropolitan art, culture

## Abstract

Recent discourse around decolonial praxis has given rise to an urgency to look again at African indigenous cultures, including Yoruba. Sadly, from the late 1600s [Ogilby’s Africa (1670)] to the 1900s, Yoruba culture was described through an outsider, ethnographic, colonial lens as primitive, ungodly, and uncivilised. Furthermore, from the early 1900s to date, due to colonialism, the Yoruba language, classed as vernacular, was prohibited from being spoken at schools in Nigeria (Oluwole, 2016). The late Dr. Geraldine Connor (1952–2011) demonstrated decolonial praxis, expanding on Homi Bhabha’s (1990) ‘third space’ and fostering a unique creative space where cultures, including Yoruba, freely mingled and equilibrated into her PhD creative output, Carnival Messiah. Theory can be bridged into Afropolitan praxis via a Practice as Research (PaR) methodology, a non-hierarchical, multimodal design that layers multiple qualitative research methods, including what the author calls ‘decolonial autoethnography’. Yoruba scholars and writers such as Nike Lawal, Sophie Oluwole, Rowland Abiodun, and Babatunde Lawal advocate that one needs to let the Yoruba voice speak as a process of decolonising one’s own mind. The aim in this decolonial praxis is to dispel negative narratives about Yoruba culture, curb anxiety about partaking in it, and experience it through the spectrum of Yoruba philosophy, in its multifaceted whole as movement, sound, sculpture, and more. Decolonial praxis, in this case, looks like observing Yoruba culture *in situ* through (vis-à-vis knowing-in-action) art practice as a process of conceptual enquiry. The main outcome of the author’s PhD project was the retrieval and resurrection of Yoruba art aesthetics, including visual representation (Aworan) and sound (Oriki). This culminated in the author’s creation of the first sculpture of a black woman in Leeds: the life-size ultramarine-blue Aworan statue of Dr. Geraldine Roxanne Connor.

## 1 Introduction

“*We are committing suicide by not speaking our language*”—Prof Sophie Oluwole.

Recent discourse around identity, the BLM movement, and #BAMEover has given rise to an urgency to look again at African indigenous cultures, including Yoruba. Leeds is rapidly growing in diversity and is home to thriving Caribbean and African communities, hosting an annual West Indian Carnival. The late Dr. Geraldine Connor was a British-Trinidadian ethnomusicologist, theatre director, and educator. Connor (2005a,b) researched Caribbean carnival culture, carnival characters, and their links to West African cultures, e.g., Yoruba culture. Connor’s Third Space of decolonial praxis allowed cultures to freely mingle and equilibrate into the creation of her PhD creative output, Carnival Messiah, a reworking of Handel’s Messiah.

Yoruba culture has also been researched and written about by Western ethnographers, historians, missionaries, and explorers, including Frobenius (1913). Yoruba culture’s artistic aesthetics were often described, from a Western, outsider, ethnographic, colonial lens, as primitive, uncivilised, or simply decorative. Christian apologists deemed Yoruba spirituality as pagan or heathen, and Yoruba speaking was prohibited in some homes and at missionary schools.

I will be asking three main questions:

How is theory bridged into praxis?

What is needed for decolonial praxis?

What does decolonial praxis look like?

### 1.1 How is theory bridged into praxis?

I am an Afropolitan visual artist, sculptor, singer, songwriter, and poet, and I utilised Practice as Research (PaR) in my PhD project. Practice as Research (Nelson, 2013), as a research methodology, is a non-hierarchical, multimodal design that imbricates and layers multiple qualitative research methods. My methods included my Carnival Messiah autoethnography case study, retrieving aspects of Yoruba culture despite prohibitions and anxieties surrounding it. Allowing the Yoruba voice to influence my art practice vis-à-vis theory, so that it sits side by side with art praxis.

### 1.2 What is needed for decolonial praxis?

Yoruba scholars and writers such as Oluwole (2015), Abiodun (2014a,b), and Lawal (2001) advocate that when studying Yoruba art aesthetics, one needs to let the Yoruba voice speak—a process of decolonising one’s own mind. Yoruba voice, put simply, means Yoruba culture sitting alongside Western colonial cultural paradigms, allowing for innovative contemporary outcomes. It also means telling Yoruba stories and narratives from an insider perspective, including speaking in Yoruba. The aim of this decolonial praxis is to dispel negative narratives about Yoruba culture, curb anxiety about partaking in it, and discover its hidden richness. Indeed, experience the culture through the spectrum of Yoruba philosophy, in its multifaceted whole as movement, sound, sculpture, and word.

### 1.3 What does decolonial praxis look like?

**Figure 1:**
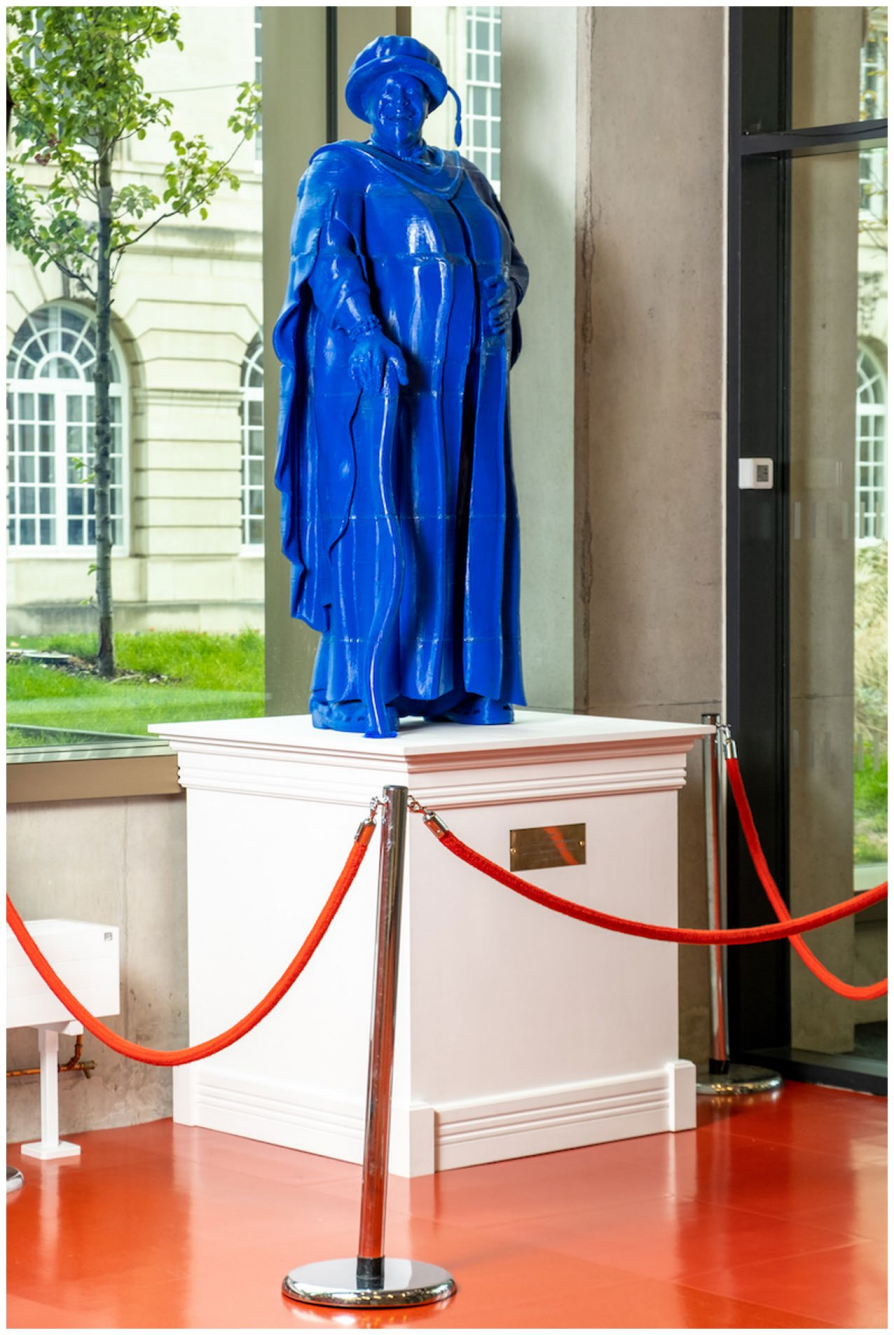
Dr. Geraldine Connor Aworan statue and first of a black woman in Leeds, UK.

Overall, decolonial praxis looks like my multimodal PhD practice as research, self-decolonising praxis of retrieving and reclaiming Yoruba culture. My discovery of Yoruba Àwòrán (visual representation) and oriki (praise singing) practices resulted in the creation of the first civic statue (Àwòrán) of a black woman in Leeds, that of Dr. Geraldine Connor (Figure 1).

## 2 A Yoruba incantation in Leeds

*Esu gbaragbo mo juba Are are.*

Ella Andall (Hickling, 2002), a Trinidadian Yoruba practitioner, cried out at the top stairwell of the Leeds Playhouse; that sounds, I thought, like Yoruba—a language we were prohibited from speaking growing up lest it pollute our English words. In my first experience of Carnival Messiah, the seeds were sown for further enquiry into a once-forbidden Yoruba culture.

### 2.1 Yoruba culture

The Yoruba people are said to have originated in Ile-Ife in present-day Nigeria, West Africa, and to have been founded by Oduduwa (Abimbola, 2006; Abimbola and Miller, 2003; Lawal et al., 2004; Ogunbitan, 2007; Usman and Falola, 2019). Yoruba is also the name of the spoken language. Yoruba culture is rich in its philosophy, artistry, and spirituality. Yoruba people and Yoruba culture have migrated voluntarily and involuntarily throughout West Africa and as far as Brazil and the Caribbean.

### 2.2 Why retrieve Yoruba culture and stories for decolonial praxis?

**Figure 2:**
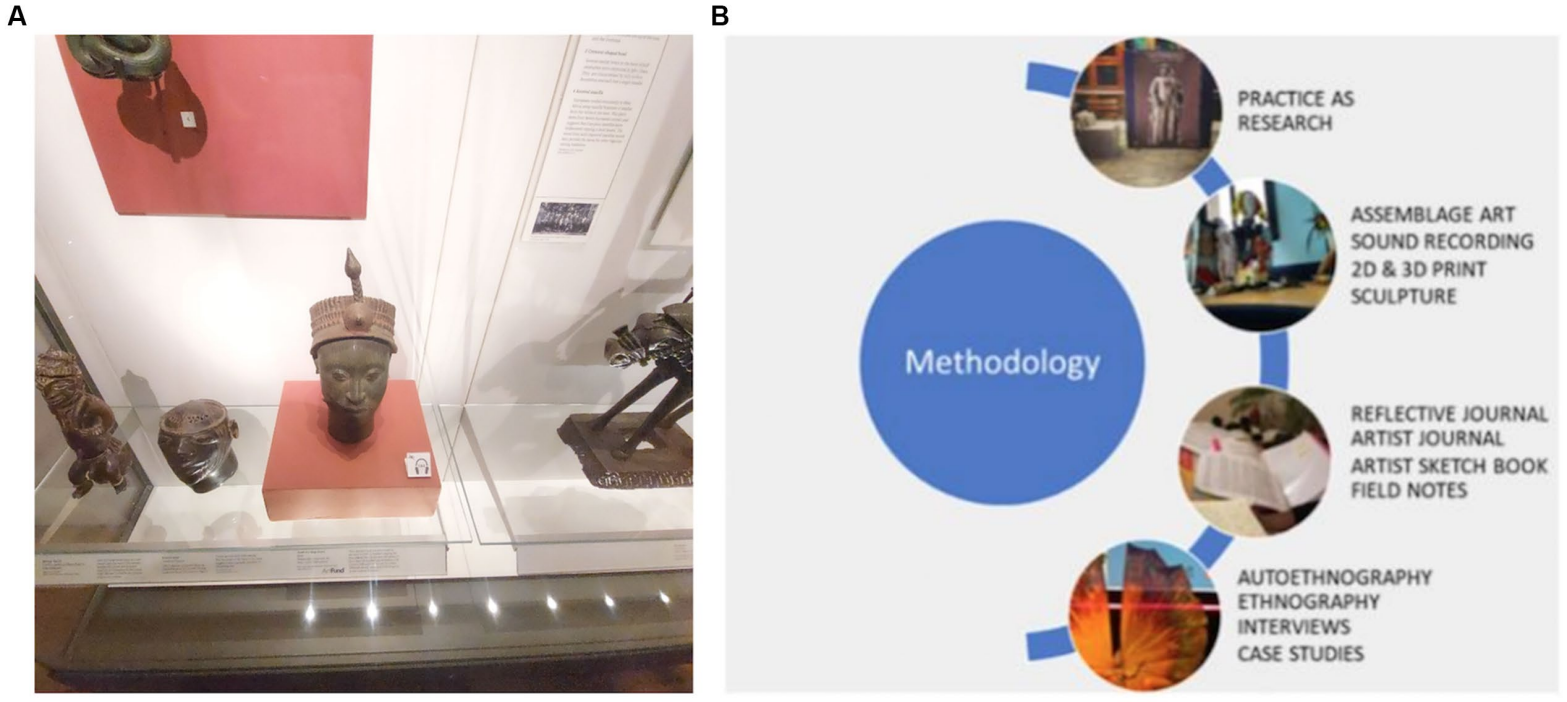
**(a)** British museum, Salisbury gallery featuring the Yoruba collection including the (Head of a king) Ori Olokun bronze statue discovered by Leo Frobenius. **(b)** How my multi modal, practice as research methodology model looks like, non- hierarchical methods feeding into each other: Doing–Thinking–Reflecting–Writing–Doing–Thinking.

Yoruba culture endured rupture, fragmentation, and disjunction in the diaspora after colonisation and the transatlantic slave trade. Yoruba artistic aesthetics were often described by colonial outsiders as primitive, uncivilised, or simply decorative. Indeed, when lifelike bronze sculptures were discovered at Ile Ife in the early 1900s, this was met with disbelief that Africans had created them (Art Documentaries, 2014). German explorer Frobenius (1913) speculated that the Head of a King (Ori Olokun) sculpture housed at the British Museum (Figure 2a, see illustrations) may have come from the lost city of Atlantis! Equally, a sculptural tradition was nearly lost after the British invasion of the Benin Empire in 1897, when countless bronzes were looted (as spoils of war) and dispersed worldwide. Thankfully, a victory for the decolonial movement is the ongoing repatriation of Benin Bronzes to Nigeria from the Ethnographical museums in Berlin and Leipzig, as well as the London Horniman Museum.

### 2.3 Linguistic colonisation—how I lost my Yoruba voice at school and home

In my TEDx Talk, *Finding my Yoruba voice* (Rose, 2025), I share how I never really had a Yoruba voice growing up at home in Nigeria and attending a missionary school in the 1980s/90s. We were prohibited from speaking any indigenous languages, including Yoruba, and it was mandatory that we speak in English; Yoruba was classed as vernacular or uncivilised. It was against school rules to speak Yoruba, and if you got caught, punishments such as stooping down, facing the wall, or writing 1,000 lines were commonplace. *I will not speak vernacular on school premises*. Similarly, at home and with relatives, communications were in English, including with my grandmother, who was a schoolteacher.

### 2.4 How is theory bridged into praxis?

Practice-as-research, as a decolonising praxis, allowed theory to sit side by side with my practice within a ‘doing-thinking-doing’ paradigm (Figure 2b, see illustrations). My practice as a research PhD involved observation, autoethnography, participation, reflective journalism, studio work, literature reviews, case studies, interviews, and conversations. Practice as research allowed me to objectively immerse myself in Yoruba culture and continuously curb my innate anxiety about forbidden aspects of the wider culture. This, in turn, influenced and informed my own artistic practice through the application of current research and the recall of fragments from memory, such as nursery rhymes and Yoruba folk songs. I reinforced my research with literature reviews (Oluwole, 2015; Abiodun, 2014a,b; Lawal, 2001) and drew direction from a few Yoruba proverbs (Owomoyela, 1981). I retrieved and explored aspects of Yoruba philosophy and Yoruba art aesthetics and gained a greater understanding of Yoruba spirituality.

For example, I discovered this proverb, which felt like a great metaphor for why the negative narratives about Yoruba culture pose challenges to overcoming the overall colonial mindset.

*Ẹni ejò bá ti bùjẹ rí, bó bá rí ekòló, yóò họ.* -Yoruba Proverb (Owomoyela, 1981).

This proverb loosely means, ‘Once beaten, twice shy’ (whoever had once been bitten by a snake would flee at the sight of an earthworm), and can be applicable to my anxiety of immersing in a once forbidden Yoruba culture. I was also greatly inspired by ancient Yoruba sculpture I had seen at the British Museum and aso-oke (high cloth), including the process of cloth weaving, and recalled masquerades such as Eyo that I had seen in Lagos, Nigeria. I wrote about my involvement in Leeds at both the West Indian Carnival and Carnival Messiah. I learnt about the ancient Yoruba sculptural practice (Abiodun, Lawal, Oluwole) of venerating ancestors and honouring people of note, which led to the creation of the Geraldine Connor civic statue.

### 2.5 Colonised ethnography vs. decolonising autoethnography—beginning to let my Yoruba voice speak

Connor (2005a,b) demonstrates linguistic and cultural decolonisation in her PhD and PhD artefact, Carnival Messiah. I feel incredibly privileged and honoured to have experienced Carnival Messiah in practice both as a performer and an observer. Carnival Messiah was a reworking of Handel’s Messiah, incorporating Caribbean, Carnival, and Yoruba culture. What I call ‘Decolonising autoethnography’ allowed me to relay my experience from an ‘insider’ perspective, rather than outdated outsider-colonising ethnographic narratives subconsciously ingrained in our psyches (Ogilby, 1670; Frobenius, 1913; Bascom, 1939). I could recall feeling a sense of homecoming and belonging while singing Yoruba lyrics alongside Handel’s classical music.

As now recognised, autoethnography is simultaneously a ‘process’ of collating research and a ‘product’ or result of the research and is described by Ellis et al. (2010, p. 1) as:

An approach to research and writing that seeks to describe and systematically analyse personal experience to understand cultural experience. This approach challenges canonical ways of doing research and representing others and treats research as a political, socially just, and socially conscious act. A researcher uses tenets of autobiography and ethnography to do and write autoethnography. Thus, as a method, autoethnography is both process and product.

As an aesthetic, ephemeral experience, Connor’s aim with Carnival Messiah was to move towards collapsing some of the essentialised differences that continued to exist between Europe, Asia, and Africa by creating a hybrid space and place where they could coexist equally. Although possibly influenced by Homi Bhabha, Connor also defined this space as the Third Space. In her accompanying thesis,*! Hallelujah! Excursions into a Third Space: Carnival Messiah as an Instrument of Postcolonial Liberation’* Connor (2005a,b, p. xi), she writes:

This new territory is what I define as the ‘Third Space’. Third Space is a space of many voices and unfixed cultural identities, the site of transformation and multi-consciousness. That moment of entry into Third Space, which in *Carnival Messiah* suspends reality, is often experienced as a moment of psychological catharsis, a moment of spiritual renewal in a short period of transported existence, even euphoria, and whose effects will last far longer than those actual moments of quintessence.

In my experience, I can attest that I indeed felt nurtured, joyous, ecstatic, and renewed during the short moments of Carnival Messiah, which have had a long-lasting effect. Connor (2005a,b, p. 3) states her ambitions for creating Carnival Messiah:

Carnival Messiah thus embodies aspirations for an integrity of being, seeking to collapse entrenched and negative notions of difference, while guiding its participants and recipients towards a critical consciousness which can only be achieved through self-discovery and self-recovery.

My autoethnography case study consisted of a recap of my experience of my own self-discovery and self-recovery as a participant and recipient of Carnival Messiah. This, in turn, boosted my confidence and served as an impetus that set me on the path of exploring Yoruba scholars crucial to decolonial praxis.

### 2.6 What is needed for decolonial praxis? African-derived paradigms from African scholars’ literature reviews

Literature reviews by Yoruba scholars, as mentioned in section 3.5 above, were needed for decolonial praxis to develop Yoruba art paradigms rather than colonial Western ones. Rowland Abiodun is a Professor of Art at Amherst College, Massachusetts (as per initial feedback). Through the Leeds Beckett University library, I was able to acquire a digital copy of his book, *Yoruba Art and Language: Seeking the African in African Art* (Abiodun, 2014a,b, p. 1), in which he states:

Art scholars rarely venture outside dominant Western paradigms, even when analysing works from non-Western cultures. In the past, this proclivity has led to an unfortunate weakness in the study of African art, as it has ignored the discovery, recognition, and analysis of African-derived paradigms.

Following on from Abiodun’s observation about the weakness of limiting academic research on Yoruba culture and art to Western paradigms and mindsets, it was crucial to discover more Yoruba paradigms, which, in turn, strengthened my research project and subsequently led to a more robust retrieval of Yoruba culture and language. Indeed, I was delighted to discover Abiodun’s paradigm of Yoruba art-making, cited below, which, in turn, led to an exponential increase in my artistic expectations and my art practice output. I went from creating masking-tape abstract mini-sculptures during the Covid-19 lockdown to embarking on the ambitious project of creating a life-size statue of Geraldine Connor! Abiodun (2014a,b, p. 282) writes:

In transforming their raw material, Yoruba artists seek to realise the ìwà (identity and essence) of their subject and se lóge (embellish) them through artistic activity, using ojú-onà (design consciousness).

I characterised this as a three-prong creative paradigm (1. ìwà, 2. se loge, and 3. ojú- onà), and I used this as an aesthetic standard to which my hybrid contemporary art practice needed to measure up to. Abiodun believes that the starting point in studying Yoruba art aesthetics is ìwà, which will not only show through but also influence an artist’s work and its execution. I realised that the intrinsic worth of things, or identity, or essence, or ìwà, needs to be fully realised in Yoruba art aesthetics, and this could be both verbal and visual. Abiodun (2014a,b, p. 254) stresses the importance of verbal and visual oríkì in recognising ìwà, stating,

The verbal and visual oríkì (*praise singing and statue – my emphasis*) of an òrìsà, human beings, and things play a vital role in the recognition of this ìwà. The oríkì constitute a powerful vehicle in the identification, expression, and realisation of the essence of everything known to the Yorùbá.

This in turn influenced my overall art aesthetic, presentation and performance to include both the visual and verbal oríkì (the visual being the statue and verbal being the praise singing)—I recently showcased this at a TEDx Talk, titled ‘Finding *my Yoruba voice*’ (Rose, 2025), where I started the talk walking in carrying a maquette statue of Geraldine on my head while singing her oríkì.

I also delved into the writings of Babatunde Lawal (2001) to deepen my understanding of Yoruba art aesthetics and to decipher why there were so many ancient bronze sculptures. Lawal is an art historian and scholar of Nigerian art and the professor of art history at Virginia Commonwealth University in Richmond, Virginia, United States. I discovered the importance of honouring community leaders and the Yoruba word for visual representation—Àwòrán. Lawal (2001, p. 498), in Àwòrán: Representing the Self and Its Metaphysical Other in Yoruba Art, writes that:

Among the Yoruba of Nigeria and the Republic of Benin, the word Àwòrán commonly refers to any two- or three-dimensional representation, ranging from the naturalistic to the stylised.

I thus adopted the word “Àwòrán” for my naturalistic 3D visual representation of the Geraldine Connor statue and others. My art practice evolved to incorporate singing, verbal oríkì, and the presentation of visual oríkì (naturalistic Àwòrán). As described by Abiodun, I wanted my contemporary art practice to meet the 3-pronged paradigm of Yoruba art-making. I believe this decolonial praxis allowed me to do this—realise the identity and essence (ìwà) of my artistic activity and fully embellish (se lóge) it with design consciousness (ojú- onà), creating the first statue of a black woman in Leeds.

## 3 Discussion: Ubuntu, linguistic decolonisation, multicultural coexistence, the third space and hybridity themes

### 3.1 Ubuntu

Fanon (1974) in ‘The Wretched of the Earth’ explains how colonisers can only go so far when colonising and, indeed, inevitably, would initiate decolonisation as the next logical outcome to save the ‘humanity’ or ‘fitness for exploration’ of both colonised and colonisers Jean-Paul Sartre writes in the opening preface that;

He ought to kill those he plunders, as they say djinns do. Now this is not possible, because he must exploit them as well. Because he cannot carry massacre on to genocide and slavery to animal-like degradation, he loses control, the machine goes into reverse, and a relentless logic leads him to decolonisation. Fanon (1974, p. 14).

“Ubuntu” is an African Zulu word that means ‘humanity to others,’ reminding us that ‘I am what I am because of who we all are.’ In other words, decolonisation to me also means re-humanising us all by embracing our multiple cultures. From an Ubuntu perspective, and to benefit all of humanity, the colonised can choose to take the reins of the decolonisation process despite it being inevitably initiated by colonisers. Nevertheless, one way the colonised can proactively include themselves in the decolonisation process is by engaging in linguistic decolonisation of African languages. Other ways include cultural transmission (e.g., syncretism) and the decolonisation of negative narratives and myths. Equally important are ‘third space’ emerging multicultural identities, hybrid art production, and educational frameworks such as Afropolitanism.

### 3.2 Ubuntu vs. cosmopolitanism

At the 2008 conference, in his presentation ‘*Cosmopolitanism: Ethics in a World of Strangers,’* Kwame Anthony Appiah, a world-leading philosopher and writer, suggests that cosmopolitanism advocates fusing universalism with the embrace of cultural differences and tolerance for one another. Appiah (2008) defines ‘cosmopolitanism’ as a Stoic philosophical ideal which, in simple terms, refers to a citizen of the world, derived from the Greek word kosmopolitēs (‘citizen of the world’). Appiah (2006) also states that, in developing cosmopolitanism for our times, he defends a vision of art and literature as a common human possession. Similarly, Turkish novelist and author of *The Bastard of Istanbul,* Shafak (2014), advocates in ‘*The Urgency of a Cosmopolitan Ideal as Nationalism Surges’* that being a cosmopolitan requires both the appreciation of hybridity, coupled with the mental/moral bridge between ‘I’ and ‘humanity’. Ubuntu and cosmopolitanism both advocate cultural tolerance and the embrace of our shared humanity, rather than elitism or colonial dominance. Afropolitanism, in turn, borrows from this cosmopolitan ideal, referring to Africans as Africans of the world.

#### 3.2.1 Expressions of the ‘third space’—syncretism, linguistic/myth decolonisation, multicultural coexistence, and Afropolitanism

Dr. Geraldine Connor, as an educator and pioneer in multicultural theatre productions, was an inspiration to the wider Leeds community, nurturing many students, and, upon discovering my Yoruba heritage, said to me, “Child, you need to tell your Yoruba story in your music and art.” Thiong'o (1986a,b) also advocates retrieving African languages and ‘decolonising the mind’ in his book titled ‘D*ecolonising the Mind: The Politics of Language in African Literature*’. Even more alarming is *Socrates and Ọ`rúnmìlà: Two Patron Saints of Classical Philosophy,* author and first female philosopher in Nigeria, Prof Sophie Oluwole (2015, 2016, 2018) fear that we are committing suicide by not speaking our language.

Thereby, the first step in decolonising praxis is a mindset shift and an ongoing process of decolonising one’s mind and, in my case, allowing my Yoruba voice and culture to speak. This can be achieved by allowing cultures to equilibrate in what Connor describes as a third space, which offers a vehicle for colonised languages and cultures, including Yoruba, to coexist alongside coloniser languages and cultures. It also offers a space for self-discovery, for curbing anxieties, and for the recovery of colonised or lost parts of the self. I had firsthand experience of Connor’s Third Space during my participation in *Carnival Messiah,* and it set me on my ongoing journey of self-recovery, homecoming, and belonging.

#### 3.2.2 My PhD multimodal methodology and hybridity themes

My PhD decolonial praxis was achieved through a practice-as-research methodology, retrieving and engaging in lost Yoruba artistic aesthetics—visual representation (Àwòrán) and verbal praise singing (Oríkì)—cultural practices alongside contemporary art practices. I utilised my discovery of Abiodun’s 3-prong artistic paradigm in my PhD study to completely realise (se logo) the ìwà (essence) of my artistic outcome (ojú onà-design consciousness)—the creation of the first statue of a black woman in Leeds, the 1.7 m life-size ultramarine blue civic Àwòrán statue of Geraldine Connor. I also coupled my research project with themes reflecting hybridity. For example, cultural transmission (syncretism), multiculturalism, cosmopolitanism, Afropolitanism, and myth decolonisation.

Connor (2005a,b) discusses four coping mechanisms that trafficked Africans had to adapt. The first, a phenomenon called ‘magical realism’, is often underrated or overlooked by Western scholarship because of its links to the spiritual or the ephemeral, in which the discourse of myth effortlessly co-exists with that of history. Within the ‘third space’, Yoruba myths and cultural survival are explored and manifest in Carnival Messiah from the outset, with libation poured to *Esu Elegbra*.

#### 3.2.3 How was Yoruba culture transmitted and retained; how did Yoruba spirituality survive in the Caribbean?

Nigerian fiction writer Owomoyela (1981, p. 126), in his African Studies journal article *The Pragmatic Humanism of Yoruba Culture,* writes that ‘it is expected that the Yoruba, a society that has endured centuries of cohesion and tension, will be found evidence of adjustments and compromises that have made its continuation possible’. Yoruba spirituality survived in the ‘third space’ of syncretism. Syncretism (syncretic amalgamation) is one such phenomenon in which different religions are combined or synthesised. Shaw and Stewart (1994) summarise in their book, *Syncretism/Anti-Syncretism: The Politics of Religious Synthesis,* that ‘syncretic amalgamation of religions may be validated as a mode of resistance to colonial hegemony, a sign of cultural survival’. Connor (2005a,b, p. 43) also suggests that syncretism is another coping mechanism, stating that:

The second is directly related to conditions of secrecy through the use of camouflage or syncretism, where, often, particularly horrific historical remembrances are portrayed/camouflaged in modern-day enactments of great aesthetic beauty or as forbidden religious practices submerged within or syncretised into the existing status quo.

#### 3.2.4 What new multicultural identities emerged?

Connor (2005a,b), as mentioned above, proposed four coping mechanisms, the last two related to identity formation, saying:

The third and fourth coping mechanisms indeed shape the basis of contemporary Caribbean identity formations. They exhibit a clear disposition toward non-violence and a predilection for a distinctive individuality that stresses plurality, hybridity, and differentiality – a unique condition that I describe as cultural multiplicity.

Cultural multiplicity within the ‘third space,’ (Bhabha, 1990; Connor 2005a,b; Wolf, 2000) in my view, also relates to the inevitable synthesis of the now-known Afropolitan identity. I believe Afropolitanism works well as both a coping mechanism and a framework for decolonial academic discourse.

#### 3.2.5 From cosmopolitanism to Afropolitanism

The term ‘Afropolitanism’ is constructed from the name “Africa” and the Greek word(s) “politis” (citizen) or, better still, the word “kosmopolitēs” (‘citizen of the world’). Contrary to popular opinion, the term “Afropolitanism” originated in South Africa, was coined by Cameroonian historian, political theorist, and philosopher Prof. Mbembe (2007), and was popularised by writer Taiye Selasi (2005). Selasi penned an essay, *Bye Bye Barber,* in which she defined Afropolitans as ‘Africans of the world’, identifying multi-local peoples of African descent worldwide. Selasi (2015), in her TED Talk, introduced the newest generation of African immigrants, saying, “Were you to ask any of these beautiful, brown-skinned people that basic question—‘where are you from?’—you’d get no single answer from a single smiling dancer.” *We are Afropolitans: not citizens, but Africans of the world!* Afropolitanism became a tool for exploring the identity and integration of diasporic peoples in ‘modern’ society, rather than through assimilation.

Professor Emeritus of Social Anthropology, Stockholm University, Hannez (2022, p. 9), in his book, *Afropolitan Horizons: Essays toward a Literary Anthropology of Nigeria,* suggests that despite all the controversy about Afropolitanism (elitism, reductive, and so on), Afropolitanism is now in the ‘domains of intellectual commons’, stating:

I would suggest that the term is now in the domain of the intellectual commons, and I will take a certain liberty with it here. Rather than engaging with ongoing debates over Afropolitanism in its current forms, mostly among literary scholars, I will use it as a term with extended time depth, more in line with Achille Mbembe’s original discussion: to sum up, with a convenient single word, the involvements of West Africa with the outside world, past and present.

Indeed, art historian and curator (Hassan, 2020a,b) delivered a keynote speech and penned an essay entitled ‘*Contemporary African Art as a Paradox: Is ‘Afropolitan’ the Answer?’* In his essay, he investigated the term ‘Afropolitan’ as a favourable innovative framework for contemporary African and African diaspora artistic or cultural production. Hassan engages with the entanglement of African culture and the West at the intersection of modernity and post-coloniality. For example, he identifies young, creative, cosmopolitan African immigrant writers such as Teju Cole, Taiye Selasi, and Chimamanda Ngozi Adichie as Afropolitans.

Hannerz (2005, p. 7) in *Two Faces of Cosmopolitanism: Culture and Politics*, states, “Cosmopolitans, ideally, would seek to immerse themselves in other cultures, participating in them, accepting them as wholes. Yet in not only embracing these cultures but also displaying their skills in handling them, there is also a sense of mastery.”

I feel that Geraldine Connor can also be classed as a cosmopolitan/Afropolitan who displays this mastery of multiple cultures within the *Third Space* in her creation of *Carnival Messiah*. To answer Hassan’s question, I believe the answer is yes: Afropolitanism is situated within the *Third Space* and is a favourable decolonial cultural hybridity framework. This also informed my decision to position myself as a Yoruba-British hybrid Afropolitan artist in my art praxis.

### 3.3 Inspiration from Geraldine’s decolonial third space

Overall, inspired by Dr. Geraldine Connor’s decolonial praxis, Afropolitanism, Lawal (2001) and Abiodun (2014a,b) Yoruba art paradigm, I began to tell my Yoruba story through the language of Oríkì and Àwòrán—verbal and visual communication and representation. My art practice utilises a mix of assemblage processes, sound, and sculpture, all informed by Yoruba culture, philosophy, and art aesthetics to tell visual stories. I use assemblage methods to create art installations, using metaphors, glue, or bandages to bind together the pieces of the African broken past and to represent difficult subjects such as the slave trade, colonialism, and racism. My assemblage installations included found objects, masking tape, sculptures, prints, sketches, photography, sound, music, poetry, and audio-visual material. My use of found objects also signified the resourceful nature of my Yoruba ancestors when they were subjected to conditions of limited physical resources.

I was also inspired by Nigerian artists Yinka Shonibare and Victor Ehikhamenor, who create assemblage installations and use hybridity in their decolonial work, incorporating elements of Yoruba culture. For example, after lockdown, Shonibare created a sculpture, *Unintended Sculpture (Donatello’s David and Ife Head)* at the Royal Academy show (2021), inspired by the *Ori Olokun* (Head of a king) sculpture and other Ife heads at the British Museum. Anthropologists like Leo Frobenius at first did not believe Africans could make such naturalistic sculptures. Royal Academy of Arts (2021) explains that anthropologists (Bascom, 1939) suggested they had discovered a *Nigerian Donatello*. In this work, Shonibare parodies Donatello by fusing the Ori Olokun head onto Donatello’s David, painted in his usual colourful African patterns. With this sculpture, he offers a revised commentary on people’s understanding of African civilisations.

I also kept reflective journals to critically appraise my practice; record my feelings; observe phenomena; review what is working and what is not; and improve my practice; decipher how to make tacit knowledge explicit, including new ideas and organising my whole project. It was during one of my reflective moments that it hit me—I felt I needed to explore the gilded realistic sculpture (Àwòrán) dug up by Leo Frobenius in 1913, and in doing so, my practice evolved with a desire to create a sculpture of Geraldine Connor and of notable people in Leeds.

I created mini-installation crown maquettes during lockdown, utilising available materials such as masking tape, wool, old packaging, and bottles. I was, however, left with a feeling of emptiness, further brokenness, anguish, and discontent the more I explored the Yoruba sculptures from Ife and Benin. I felt I needed to do Yoruba culture justice (*se logo*) in my art practice by honouring notable community members with actual Yoruba Àwòrán. It became clear that honouring Dr. Geraldine Connor would mean creating such a befitting Àwòrán as would be in keeping with Yoruba culture.

Excitedly, after a tedious process of researching how to achieve this in the middle of lockdown and without access to the workshops, I remotely utilised current 3D design and printing technology [or design consciousness (ojú onà)] to create the first maquette of Connor from found photographs. I chose blue for the final scaled-up lifesize statue to represent the Carnival Messiah ‘Mama god’ character, inspired by Yoruba sea/ocean deities, Olokun and Yemoja, respectively. I sometimes pair presenting the statue with a verbal performance about Geraldine’s accolades, combining Yoruba and English lyrics and incorporating the Yoruba opening lyric from Carnival Messiah.

As mentioned above, upon rediscovering the ancient Àwòrán practice in Yoruba culture, I felt it was very important to enshrine Geraldine as an Àwòrán so future generations could be introduced to her and inspired by her. Geraldine’s decolonial artistic and academic legacy, the Third Space blueprint, and the joyous rapture of her multiple audiences’ experience of Carnival Messiah are now visually enshrined in the blue colour of the 1.7 m statue (Àwòrán), the PhD regalia, and the expression of joy captured in her face!

I conclude with Geraldine Connor (2005a,b, p. 377) words, highlighting the importance of creating art from within the Third space (blueprint/paradigm), what Carnival Messiah meant to her, and why I believe her legacy needs to live on for humanity as a whole and in the spirit of Ubuntu:

This Third Space encourages sight from new perspectives, the elimination of boundaries between margin and centre, subversions, transgressions, and the creation of a new universe where all differences are to be affirmed and celebrated. Carnival Messiah thus becomes the embodiment of aspirations toward achieving integrity of being, collapsing entrenched and negative notions of difference while guiding its participants and recipients towards the attainment of a critical consciousness which can only be achieved through self-discovery and self-recovery. Within this Third Space Carnival, Messiah strives to exemplify forgiveness, reconstruction, self-affirmation, healing, and unification as an allegory that enables transformation and enlightenment.

## Data Availability

The original contributions presented in the study are included in the article/supplementary material; further inquiries can be directed to the corresponding author.

## In memoriam

This article is dedicated to the memory of Dr Geraldine Connor, inspiring me to tell my Yoruba story. I am forever grateful for your motherly support, emotionally, artistically, and spiritually.
